# Recovery in Stroke Rehabilitation through the Rotation of Preferred Directions Induced by Bimanual Movements: A Computational Study

**DOI:** 10.1371/journal.pone.0037594

**Published:** 2012-05-24

**Authors:** Ken Takiyama, Masato Okada

**Affiliations:** 1 Graduate School of Frontier Sciences, Department of Complex Science and Engineering, The University of Tokyo, Chiba, Tokyo, Japan; 2 RIKEN Brain Science Institute, Wako, Japan; Hôpital Robert Debré, France

## Abstract

Stroke patients recover more effectively when they are rehabilitated with bimanual movement rather than with unimanual movement; however, it remains unclear why bimanual movement is more effective for stroke recovery. Using a computational model of stroke recovery, this study suggests that bimanual movement facilitates the reorganization of a damaged motor cortex because this movement induces rotations in the preferred directions (PDs) of motor cortex neurons. Although the tuning curves of these neurons differ during unimanual and bimanual movement, changes in PD, but not changes in modulation depth, facilitate such reorganization. In addition, this reorganization was facilitated only when encoding PDs are rotated, but decoding PDs are not rotated. Bimanual movement facilitates reorganization because this movement changes neural activities through inter-hemispheric inhibition without changing cortical-spinal-muscle connections. Furthermore, stronger inter-hemispheric inhibition between motor cortices results in more effective reorganization. Thus, this study suggests that bimanual movement is effective for stroke rehabilitation because this movement rotates the encoding PDs of motor cortex neurons.

## Introduction

One of the challenges of rehabilitation research is to elucidate efficient method of promoting the functional recovery of upper limb movement in stroke patients. In neuroscience, a related challenge is determining the neural mechanisms of such functional recovery. Although stroke patients tend to recover lower limb movement after therapeutic intervention, the majority of these patients (65%) do not regain full movement of their upper limbs [1]–[3]. Recent studies have suggested that patients can recover the use of paretic upper limbs through several therapeutic methods such as constraint-induced therapy [1], [4], in which patients are restricted to using only the paretic arm by immobilizing the healthy arm. Although stroke patients can recover upper limb movement after this therapy, the neural mechanisms of this recovery remain unknown.

A recent computational study suggested that constraint-induced therapy is effective because it leads to the reorganization of a damaged region in the motor cortex based on supervised and unsupervised learning [1]. The results of this computational study thus explain several aspects of the observed effects of rehabilitation; e.g., stroke patients recover upper limb movement only when they undertake more than a threshold number of rehabilitation trials [1], [5]. Therefore, a computational approach will likely be effective for determining the neural mechanisms of functional recovery in recovered patients.

Although previous computational studies investigated the unimanual movements of stroke patients, individuals often move their arms bimanually. Bimanual movement is effective for the recovery of paretic arm movement [6]–[8]; i.e., bimanual movement facilitates recovery and retention of the recovery effect. However, it is unknown why bimanual rehabilitation is effective for stroke rehabilitation. It remains unclear what differences between unimanual and bimanual movement result in the effectiveness of bimanual rehabilitation. This study approaches this question using a computational model inspired by neurophysiological results related to bimanual movement.

Neural activities in the motor cortex differ during unimanual and bimanual movement [9]–[11]. In unimanual movements, when subjects move their right arms towards a radially distributed target, neural activity in the left motor cortex can be well fit by the cosine function of the target angle [12], indicating that each neuron is maximally activated when subjects reach in the preferred direction (PD) of the neuron and that neural activity is determined by the movement direction of the contralateral arm. Although neural activity is assumed to be influenced only by contralateral arm movement, this activity is also influenced also by ipsilateral arm movement [9]–[11], [13]. Because bimanual movement is a combination of right and left arm movements, neural activity in bimanual movement appears to be well fit by a linear summation of the activities in ipsilateral and contralateral arm movement. However, this linear summation cannot explain the neural activity in bimanual movement [9]–[11]. Furthermore, the PD and modulation depth (height of the fitted cosine function) of each neuron are different in bimanual and unimanual movement. In bimanual movement, motor cortex neurons are not maximally activated when subjects move their contralateral arms in the PD determined in unimanual movement. Thus, these differences in neural activity may explain why bimanual movement is effective for stroke rehabilitation.

Using a computational model of stroke rehabilitation [1], we investigated the following two questions: 1) what type of changes in bimanual movement affected stroke recovery or the reorganization process of the damaged motor cortex and 2) when was bimanual rehabilitation strongly effective for the reorganization process? First, we demonstrated that bimanual rehabilitation is effective because this rehabilitation causes changes in PD; changes in PD rather than in modulation depth provide a neural mechanism for the effectiveness of bimanual rehabilitation for motor cortex reorganization. Additionally, we observed the effectiveness of bimanual rehabilitation only when the PD changes were in encoding but not in decoding. Second, we confirmed that bimanual rehabilitation is strongly effective when the encoding PDs are strongly rotated. On the basis of a previous computational study [10], the present study hypothesized that bimanual rehabilitation was strongly effective when the encoding PDs undergo large changes.

## Results

This study investigated how bimanual movement affected stroke recovery by modeling the different PDs and modulation depths during unimanual and bimanual movement. During bimanual movements, we invesitigated only bimanual parallel movements in which the right and left arms move in the same directions, because the neural activities during these parallel bimanual movements have been investigated previously. We describe the definitions used in the model in detail in the *Methods* section.

**Figure 1:**
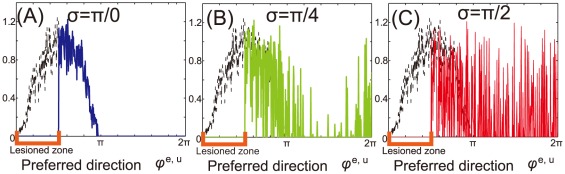
Neural activities in unimanual and bimanual movement after stroke. Dotted and solid lines denote the neural activities before and after stroke, respectively, when 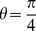. (A): Solid lines indicate neural activities when 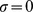. (B): Neural activities when 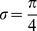. (C): Neural activities when 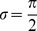.

Initially, in simulating bimanual rehabilitation, we assume that changes occur only in the PDs. These changes are referred to as PD rotations for the remainder of this manuscript. Because PDs are rotated pseudo-randomly in bimanual movement, we modeled these rotations as
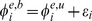
(1)where 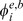 and 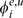 are the encoding PDs in bimanual and unimanual movements, respectively (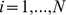), and 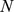 is the number of neurons. The encoding PD determines the cosine-tuned neural activity, 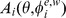, where 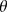 is the angle of a reaching target and the index 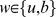 indicates unimanual or bimanual movement (see the *Methods* section). The encoding PDs are rotated 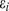 degree that is randomly sampled from a Gaussian distribution with a mean of 0 and a variance of 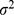. Additionally, these PDs are rotated using quenched random variables, meaning that 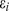 is invariant across trials. In contrast to quenched random variables, the encoding PDs can be rotated using annealed random variables sampled from trial to trial, but this type of rotation cannot facilitate the reorganization of a damaged motor cortex (see the *Discussion* regarding the effects of the annealed random variables on the reorganization). Due to the rotations of the encoding PDs, neural activities are different in unimanual and bimanual movement as shown in figure 1. In the subsection titled *Importance of encoding PD rotations for reorganization*, we consider changes of modulation depth, i.e., 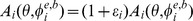, but the rotations of encoding PDs are primarily investigated.

Decoding PDs are used to calculate the population vector (PV) as follows:
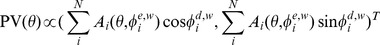
(2)with a direction and amplitude that model the direction and velocity of the reaching movements, respectively [12], [14], [15], and 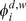 is the decoding PD. In contrast to the encoding PDs that determine neural activities, decoding PDs determine movement directions; i.e., the 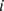
th neuron generates the motor command for moving arms in the direction 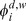. Following previous studies [14], [16], [17], in our simulations of unimanual movement, the decoding PD is set to equal the encoding PD (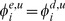). During bimanual movement, we assume that decoding PDs are not rotated on the basis of previous studies [10] (but see [11]), but in the section *Importance of encoding PD rotations for reorganization*, the modeling of rotations in decoding PDs is described.

We can model the upper limb movements of stroke patients using a PV by removing a fraction of the model neurons [1], [18] (see also [19]). Because stroke patients have difficulty in reaching in a particular direction [20], we depleted 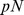 neurons with encoding PDs of approximately 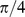, where 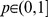 is the fraction of depleted neurons. We refer to 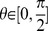 and other 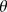 as the movement directions of large and small errors, respectively. For this damaged motor cortex, stroke rehabilitation induced a reorganization that can be modeled using an optimization framework. In this framework, the rehabilitation modifies 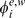 to minimize the cost function
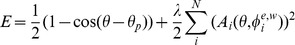
(3)where 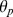 is the angle of the PV and 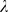 is the regularization parameter [1]. We model stroke rehabilitation using two optimization terms, supervised and unsupervised learning of 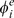, which coincide to the first and the second terms in equation (3), respectively. In the rehabilitation trials, patients moved their arms towards one of eight radially distributed targets (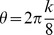, 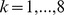) that are selected with the same probability, i.e., 
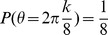
. After each rehabilitation trial, supervised learning resulted in decreased movement error between the PV and the target angle. Stroke rehabilitation also decreased the metabolic cost of neuronal activity, which was modeled by the unsupervised learning.

### Reorganization due to bimanual movement

**Figure 2:**
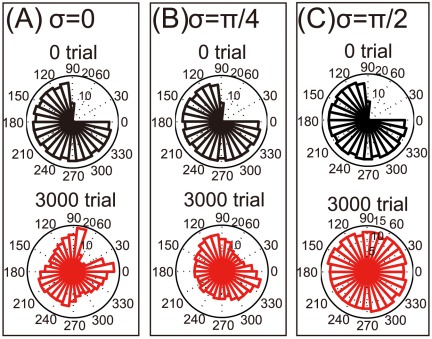
Reorganization of a damaged motor cortex after unimanual and bimanual rehabilitation (A): Histograms of 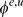 when 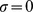. (B): The same histograms when 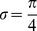. (C): The same histograms when 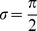.

After a neuronal lesion, we investigated whether reorganization in the damaged motor cortex can be facilitated by rotations of the encoding PDs. When the encoding PDs are not rotated (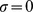), i.e., in unimanual rehabilitation, these PDs are reorganized to increase the number of neurons with encoding PDs close to the movement directions of large errors (figure 2A). In agreement with a previous study [1], however, the encoding PDs cannot be concentrated only in just these directions, especially in the middles of these directions (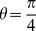). By contrast, when the encoding PDs are rotated (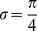 or 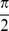), i.e., in bimanual rehabilitation, the encoding PDs are reorganized to localize in the depleted region (figures 2B and 2C). Comparing figures 2B and 2C, larger rotations of the encoding PDs lead to better the equalization of these PDs. In the *Discussion* section, we discuss the conditions in which encoding PDs are strongly rotated. Taken together, in both unimanual and bimanual rehabilitation, encoding PDs are reorganized after the neuronal lesion, but these PDs are only reorganized in the movement directions of larger errors after bimanual rehabilitation.

### Behavioral effects of bimanual rehabilitation

**Figure 3:**
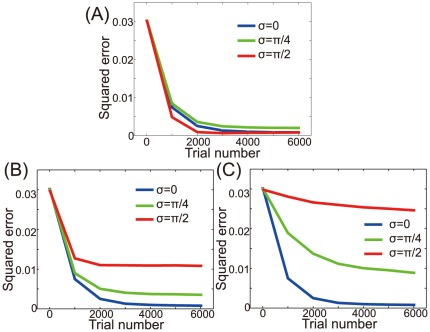
Angular error between the target and PV in unimanual movement. (A): The angular error between the target position and PV when the encoding PDs were rotated in bimanual movement. (B): The angular error when only modulation depth was changed in bimanual movement. (C): The angular error when both encoding and decoding PDs were rotated in bimanual movement.

Bimanual rehabilitation facilitates the reorganization of damaged motor cortex, but it remains unknown how this rehabilitation affects behavioral aspects such as movement error and speed. Based on the PV model, we investigated these movement parameters in unimanual reaching after either unimanual or bimanual rehabilitation. Both unimanual and bimanual rehabilitation decreased the angular error between the target position and the PV (figure 3), suggesting that these rehabilitations can restore movement precision. Bimanual rehabilitation allows angular error to reach its minimum value when 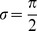, indicating that when encoding PDs are strongly rotated, bimanual rehabilitation enhances movement precision.

**Figure 4:**
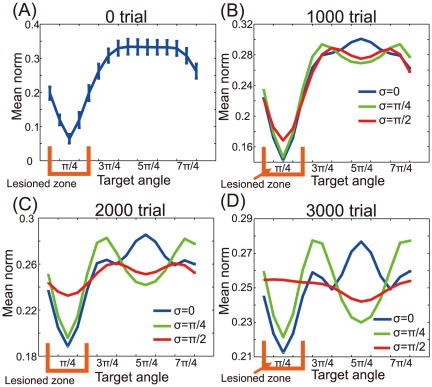
Norm of the PV after rehabilitation. (A): The norm of the PV immediately after the neuronal lesion. (B): The norm of the PV after 1000 rehabilitation trials. (C): The norm of the PV after 2000 rehabilitation trials. (D): The norm of the PV after 3000 rehabilitation trials.

In addition to movement error, we investigated how unimanual or bimanual rehabilitation affects movement speed by calculating the norm of the PV (figure 4). After the neuronal lesion, reaching speed becomes critically slower in the directions of large error (figure 4A). With moderate rotations of the encoding PDs (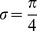), bimanual rehabilitation improved reaching speed in the movement directions of larger errors; however, this rehabilitation also resulted in reduced reaching speed in the directions of small errors (figures 4B, 4C). With strong rotations of the encoding PDs (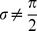), bimanual rehabilitation effectively improved reaching speed in the movement directions of large errors without hindering speed in those directions of small errors, suggesting that bimanual rehabilitation can be strongly recommended when bimanual movement induces strong rotations of the encoding PDs.

### Importance of the rotation of encoding PDs on reorganization

**Figure 5:**
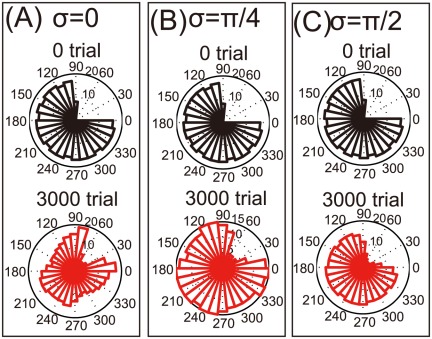
Reorganization of the damaged motor cortex when only modulation depth was changed in bimanual movement. (A): Histograms of 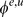 when 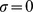. (B): The same histograms when 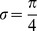. (C): The same histograms when 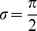.

**Figure 6:**
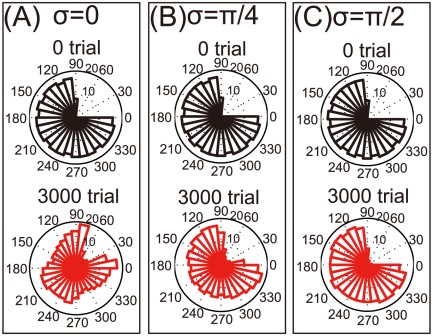
Reorganization of the damaged motor cortex when both encoding and decoding PDs were rotated in bimanual movement. (A): Histograms of 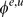 when 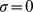. (B): The same histograms when 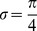. (C): The same histograms when 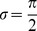.

This study originally modeled the rotations of encoding PDs, but we remained unsure whether these rotations are important for the reorganization of a damaged motor cortex. Bimanual movement causes changes not only in PDs but also in modulation depth, and decoding PDs may also be rotated. First, we simulated changes in modulation depth without PD rotations (figure 5); we observed no reorganization after bimanual rehabilitation. Next, we simulated the rotations of both encoding and decoding PDs, i.e., 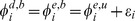 (figure 6); however, after bimanual rehabilitation, we again observed no reorganization. Thus, we concluded that the rotation of encoding PDs, as opposed to changes in either modulation depth or decoding PDs, is the most important factor in reorganization via bimanual rehabilitation.

### Nonuniform distribution of the target position

**Figure 7:**
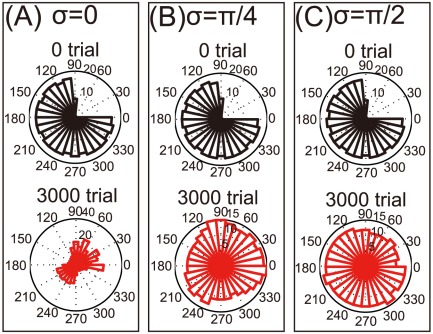
Reorganization of the damaged motor cortex when limited targets were presented in rehabilitation trials. (A): Histograms of 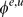 when 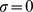. (B): The same histograms when 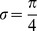. (C): The same histograms when 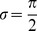.

In the aforementioned results, motor cortex reorganization was investigated by presenting one of the eight targets with equal probability, but it remains unclear whether the reorganization still occurs when only limited targets are presented. Intuitively, this reorganization appears to effectively occur when patients repeatedly move their arms in the movement directions of large errors. We simulated this case for both unimanual and bimanual rehabilitation. In unimanual rehabilitation, excess reorganization occurred in the movement directions of larger errors, with a decrease in the number of neurons with encoding PDs approximately aligned with the directions of small errors (figure 7A). When patients did not practice reaching in the directions of large errors, effective reorganization did not with either unimanual or bimanual rehabilitation (data not shown). By contrast, in bimanual rehabilitation, uniform reorganization occurred despite a nonuniform target distribution (figures 7B and 7C). Even when a limited number of targets were presented, reorganization was facilitated during bimanual rehabilitation.

### Effect of the learning rule on reorganization

**Figure 8:**
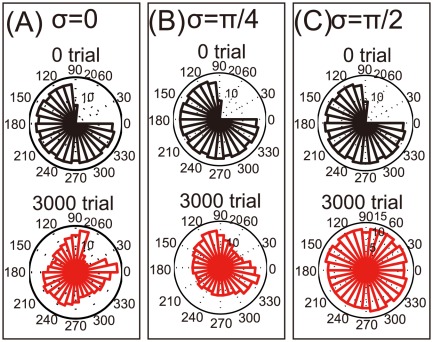
Reorganization of the damaged motor cortex with only supervised learning. (A): Histograms of 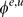 when 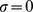. (B): The same histograms when 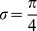. (C): The same histograms when 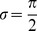.

**Figure 9:**
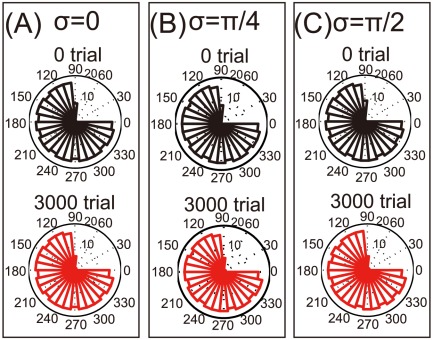
Reorganization of the damaged motor cortex with only unsupervised learning. (A): Histograms of 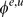 when 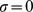. (B): The same histograms when 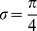. (C): The same histograms when 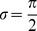.

Reorganization occurs due to both supervised and unsupervised learning, but it is unclear which learning rules better facilitates reorganization. With only an unsupervised learning rule, the reorganization was not facilitated independently of whether encoding PDs are rotated (figure 8). Conversely, when the only a supervised learning rule, the reorganization is facilitated when encoding PDs are rotated (figure 9). In bimanual rehabilitation, the reorganization is thus facilitated mainly by supervised learning.

## Discussion

After a neuronal lesion, reorganization of a damaged motor cortex can be facilitated by the rotations of the encoding PDs; these rotations are induced by bimanual movement (figure 2). Moderate reorganization can increase the reaching velocities of movements in the directions of large errors but decrease the velocities in the directions of small errors (figure 4B). However, when encoding PDs are strongly rotated, the reorganization facilitates the recovery of reaching velocities in the directions of large errors without decreasing the velocities in the directions of small errors (figure 4C), which effectively facilitates the recovery of reaching precision (figure 3). The reorganization occurs only due to the rotations of encoding PDs (figures 2, 5, and 6), suggesting that bimanual rehabilitation is effective for stroke recovery because this movement induces the rotation of encoding PDs. We also conclude that bimanual rehabilitation is strongly effective when bimanual movement induces strong rotations of encoding PDs.

Because there are few hypothesis-driven studies in this field [3], to our knowledge, our conclusions are likely the first hypotheses regarding bimanual rehabilitation in stroke patients. However, we should note in what conditions encoding PDs are strongly rotated and in which cases bimanual rehabilitation is thus strongly recommended. A previous computational study suggested that stronger inter-hemispheric inhibition results in stronger rotation of the encoding PDs for the following reason [10]. In unimanual movement of the right arm, the encoding PD determines the tuning curve of the 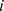
th motor cortex neuron in the left hemisphere as 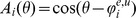. For simplicity, we neglected nonlinearity and neural noise here. In bimanual movement, motor cortex neurons in the right hemisphere are also activated; these neural activations excite the inhibitory interneurons in the left hemisphere through excitatory corpus callosum connections. These interneurons inhibit the 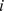
th neuron in the left hemisphere as follows:
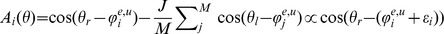
where 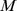 is the number of right-hemispheric neurons projecting to the inhibitory neuron and 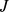 is the strength of inter-hemispheric inhibition determined by both the corpus callosum connections and the connections between interneurons and the 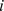
th neuron. Although the encoding PDs are not rotated (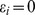) when 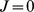, stronger the inter-hemispheric inhibition yields a stronger rotation of the encoding PDs. These PDs are randomly rotated only when inter-hemispheric connectivities are sparse; i.e., when 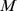 is sufficiently large, 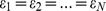 due to the law of large numbers. In actual rodent and primate brains, callosal connectivities are sparse in the hand region of primary motor cortex [21], [22]. The strength of inter-hemispheric inhibition can be estimated from imaging data such as functional magnetic resonance imaging data [23]. In summary, our computational study suggests that we can strongly recommend bimanual rehabilitation for patients with inter-hemispheric inhibition that is stronger than a specific threshold value. Although several previous studies have suggested that unimanual and bimanual rehabilitation affect stroke recovery equivalently [3], based on our results, we observed that bimanual rehabilitation facilitates stroke recovery only when inter-hemispheric inhibition is strong. Thus, we must determine the threshold inhibition value in a future project.

Although this study distinguished between encoding and decoding processes, it is unclear why exactly we are able to separate these processes. Encoding and decoding processes are equivalent to motor planning and execution, respectively. In motor planning, the neural activities of motor cortex neurons are determined on the basis of the position of the presented target. In motor execution, these neurons send motor commands to muscles through the spinal cord. Based on a study by Georgopoulos et al. [12], because the PV models actual reaching movements well, motor cortex neurons have encoding PDs that are likely equal to or very close to decoding PDs in unimanual movement. This symmetry between encoding and decoding PDs may be broken when a perturbation is applied to the reaching movement. When adapting to a visuomotor rotation or force field, neural activities change to minimize the error between target position and actual movement. When adapting to a visuomotor rotation, neural activities change only in the motor planning phase [24], suggesting that only encoding PDs change to minimize the error. During force field adaptation, a recent computational model separately modeled encoding and decoding processes; when only the encoding process is adaptable for error minimization, this motor cortex model can explain the experimental neurophysiological data of the motor cortex [25]. To our knowledge, there is little evidence regarding whether encoding and decoding processes should be separated in bimanual movement. However, we believe that these processes should be separated on the basis of the aforementioned studies of unimanual movement.

Motor cortex neurons send motor commands to muscles [26], and thus, we should note the relationship between PDs and muscles. To our knowledge, the mechanism for the determination of encoding PDs is unknown; however, based on the PV frameworks, encoding PDs are likely to be similar to decoding PDs in unimanual movement [12]. Contrastingly, using a realistic biomechanics model, decoding PDs are suggested to be determined by the strength of the connectivities between a neuron and each muscle [27], [28], i.e., the connectivities modeling cortical-spinal-muscle or direct cortical-muscle connections. When a neuron has strong connections to elbow extensors and weak or negative connections to elbow extensors, this neuron has a decoding PD in the upper-right direction in the horizontal plane. Based on these computational frameworks, a decoding PD is thus determined by the degree to which the neuron is connected to agonists or antagonists in the assumed movements. These previous studies assumed only unimanual movement, and thus, it remains for future research to determine how biomechanical properties affect decoding PDs in bimanual movement. Based on our assumptions, decoding PDs are determined in accurately generating both unimanual and bimanual movements, and rotations of the encoding PDs are responsible for generating the different motor commands in unimanual and bimanual movement. In our framework, we must thus define a biomechanics model in subsequent work.

Rotations of the encoding PDs facilitate cortical reorganization, but it remains unclear why this reorganization is facilitated by these rotations. Because we concentrated on the recovery of feedforward movements in this study, the reorganization occurs to minimize the angular error. After 3000 trials of unimanual rehabilitation, the reorganization decreased the angular error when reaching in the movement directions of larger errors by reorganizing neurons to localize in these directions. These decreases occurred because, for example, even when there is a small neuron with an encoding PD of only 45 degrees, the average PV across many trials can be directed towards 45 degrees when the neurons are reorganized as an equivalent number of neurons with encoding PDs of approximately 30 (45−15) and 60 (45+15) degrees. However, in this case, the norm of the PV is smaller than that of the well-equalized population; this reorganization is observed when the encoding PDs are strongly rotated. Furthermore, in well-equalized populations, more neurons are activated in generating PVs oriented at 45 degrees, and movement precision is better than that observed in the impaired populations observed after unimanual rehabilitation. In other words, in unimanual rehabilitation, the reorganization stops at one of the local solutions for which the angular error is decreased to some extent, but movement speed does not recover. When non-quenched (trial-by-trial variant) random variables are added to each rehabilitation (learning) step, these variables act as search noise and cause reorganization, thus avoiding trapping in local solutions [29]. As described in the next paragraph, it remains unclear whether such random variables are available in rehabilitation trials, but strong and quenched rotations may play similar functional roles. Thus, we suggest that strong rotations of the encoding PDs play the role of search noise, enabling the reorganization to avoid local solutions and leading the damaged motor cortex to a global solution, i.e., a well-equalized population.

**Figure 10:**
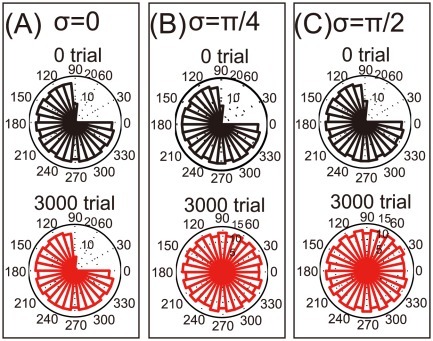
Reorganization of the damaged motor cortex when annealed noise was added to each learning step. (A): Histograms of 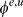 when 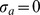. (B): The same histograms when 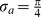. (C): The same histograms when 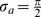.

This study investigated the influence of quenched (trial-by-trial invariant) rotations of encoding PDs on reorganization, but annealed (trial-by-trial variant) rotations can also be considered. However, we observed no significant reorganization when the rotations were annealed (data not shown). By contrast, when we added annealed noise to each learning step (see *Methods*), i.e., when we considered synaptic drift, we observed significant reorganization (figure 10). Additionally, even when the strength of this synaptic drift was moderate, we observed significant reorganization, equivalent to that observed in the case of strong and quenched rotations (figure 10B), suggesting that moderate synaptic drift enables the avoidance of local solutions and leads to a global solution. Despite the effectiveness of annealed rotations on reorganization, it remains unclear whether synaptic drift occurs during the reorganization process [30] (but see [25]). Conversely, quenched rotations likely occur in bimanual movement [10]. Our computational study suggests that strong and quenched PD rotations can effectively facilitate reorganization, but in future work, we plan to investigate how synaptic drift can be parsimoniously induced.

## Methods

### Definitions

This study assumed the following conditions: subjects move their arms towards one of eight radially distributed targets at angles of 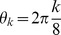 (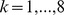), and the reaching movements are modeled as weighted averaging of neural activities. If the 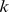
th target is presented, then the 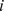
th neuron is activated as follows:
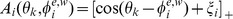
(4)where 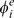 is a uniformly distributed encoding PD (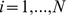), 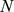
(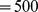) is the number of model neurons, the index 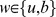 indicates unimanual or bimanual movement, and 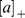 denotes a rectified nonlinearity of neural activity, where 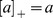 if 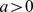 and 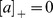 if 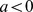. The neural activity is noisy due to signal-dependent noise, 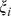, has a mean of 0, and its variance is determined by
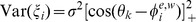
(5)where 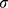
(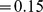) is the strength of the noise.

Based on these neural activities, the neural population generates a population vector 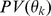 defined as
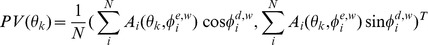
(6)where 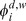 is the decoding PD. Following previous studies, the decoding PD is set to equal the encoding PD in unimanual movement (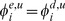) [1], [12]. Although the PV is normalized by the summation of neural activity in the standard PV algorithm, this study assumed normalized neural activity, which allows the PV to be normalized simply by using a weighted average of the neural activities, as shown in equation (6) [1].

#### Stroke implementation

The PV can be used to model the reaching movements of stroke patients by depleting a fraction of the model neurons [1], [18]. As an initial condition, we removed model neurons with encoding PDs of approximately 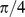 because stroke patients have difficulty moving their paretic limbs to targets far from their body centers [20]. We thus removed the neurons for which 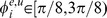. We modeled the recovery process by defining supervised and unsupervised learning (see the following section) because these learning rules model the recovery process of post-stroke rehabilitation.

#### Stroke recovery implementation

After the neuron depletion we modeled the reorganization process of a damaged motor cortex as the optimization process of the following cost function:
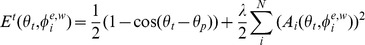
(7)where 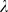 is the regularization parameter and 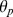 is the angle of PV at the 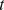
th trial. The reorganization process decreases the angular error between 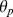 and 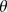 by rotating PVs toward the target position; this supervised learning is modeled by the first term of equation (7). The process also decreases the total neural activation, or metabolic cost; this unsupervised learning is modeled by the second term of equation (7). After each rehabilitation trial, the reorganization occurs as follows:
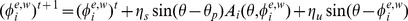
(8)where 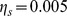 and 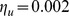 are the learning rates for the supervised and unsupervised learning methods, respectively. The learning process is not affected by these learning rates, as shown in figures 8 and 9. The reorganization is facilitated when this learning step includes synaptic drift (figure 10); this drift is modeled by adding a random Gaussian variable 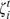 to this learning rule, for which the variable mean is 0 and the variance is 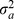.

#### Bimanual movement implementation

We assumed that bimanual movement induces rotations of the PDs only during encoding and not in decoding [10] (but see [11]). In other words, we assumed that bimanual movement changes neural activities through inter-hemispheric inhibition between primary motor cortices, but this movement does not affect cortical-spinal-muscle connectivities. In bimanual movement, the encoding PDs are rotated pseudo-randomly [10]; these rotations are modeled as 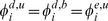 and 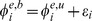, where 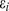 is a random Gaussian variable with a mean of 0 and a variance of 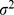. Additionally, 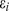 is a quenched random variable, meaning that this variable is invariant across trials; we assumed constant rotations of the encoding PDs in bimanual movement.
